# Pregnancy Differentially Regulates the Collagens Types I and III in Left Ventricle from Rat Heart

**DOI:** 10.1155/2014/984785

**Published:** 2014-07-24

**Authors:** Sarai Limon-Miranda, Diana G. Salazar-Enriquez, Jesus Muñiz, Mario V. Ramirez-Archila, Enrique A. Sanchez-Pastor, Felipa Andrade, Jose G. Soñanez-Organis, Edgar F. Moran-Palacio, Adolfo Virgen-Ortiz

**Affiliations:** ^1^Division of Science and Engineering, Department of Chemical Biological and Agropecuary Sciences, University of Sonora, Lazaro Cardenas 100, Colonia, Campus Navojoa, Francisco Villa, 85890 Navojoa, SON, Mexico; ^2^University Center for Biomedical Investigations, University of Colima, 28045 Colima, COL, Mexico; ^3^Technological Institute of Colima, 28976 Villa de Alvarez, COL, Mexico

## Abstract

The pathologic cardiac remodeling has been widely documented; however, the physiological cardiac remodeling induced by pregnancy and its reversion in postpartum are poorly understood. In the present study we investigated the changes in collagen I (Col I) and collagen III (Col III) mRNA and protein levels in left ventricle from rat heart during pregnancy and postpartum. Col I and Col III mRNA expression in left ventricle samples during pregnancy and postpartum were analyzed by using quantitative PCR. Data obtained from gene expression show that Col I and Col III in left ventricle are upregulated during pregnancy with reversion in postpartum. In contrast to gene expression, the protein expression evaluated by western blot showed that Col I is downregulated and Col III is upregulated in left ventricle during pregnancy. In conclusion, the pregnancy differentially regulates collagens types I and III in heart; this finding could be an important molecular mechanism that regulates the ventricular stiffness in response to blood volume overload present during pregnancy which is reversed in postpartum.

## 1. Introduction

Cardiovascular and hormonal changes are induced during pregnancy, which include a marked increase in cardiac output, heart rate, blood volume, and stroke volume and a decrease in total vascular resistance [1–3]. In rats, cardiomyocytes are undergoing a metabolic remodeling in late pregnancy [4]. In human and animals (rats and mice), it has been reported that a prolonged volume overload during pregnancy triggers eccentric cardiac hypertrophy which is reversible in postpartum. This hypertrophy is the result of individual growth of cardiac myocytes, predominantly in the longitudinal axis [5, 6]. The molecular signaling pathways in pathological cardiac hypertrophy and cardiac remodeling have been widely studied; however, the physiological cardiac remodeling induced by pregnancy and the reversion in postpartum are poorly understood.

The molecular signaling implicated in pregnancy-induced cardiac hypertrophy has been studied by different authors [5, 7–10]; however, there is little information about the remodeling of cardiac extracellular matrix during pregnancy. The extracellular matrix is a fundamental structure for cardiac function and is composed mainly of collagen, which contributes to passive tension in stretched cardiac muscle, thereby influencing the end-diastolic volume of the heart [11, 12]. The cardiac stiffness is associated to the expression of collagen isoforms, mostly type I collagen (Col I) and type III collagen (Col III) [13]. Col I is well-structured fibers that resist extension, while Col III forms a fine reticular network that is more compliant [14]. In clinical studies, the increase in both collagen content and Col I/Col III ratio leads to reduced myocardial compliance and diastolic dysfunction, which can result in heart failure [15, 16]. Despite its clinical importance, the adaptive remodeling in collagen isoforms expression in heart during pregnancy has not been elucidated.

In the present study, we investigated changes in Col I and Col III mRNA and protein during pregnancy and postpartum in rat heart. Our data could help to understand the molecular mechanisms involved in the remodeling of cardiac extracellular matrix during pregnancy and postpartum.

## 2. Materials and Methods

### 2.1. Animals

Animal care and experimental procedures were approved by the Ethics Committee of the University of Colima, using guidelines based on the Guide for the Care and Use of Laboratory Animals (US Department of Health, NIH). Three-month-old female Sprague-Dawley rats were separated into four groups: controls or nonpregnant rats (NP, diestrus 260 ± 8 g, *n* = 10), midpregnancy rats (MP, 12 days of gestation, 290 ± 10 g), late-pregnancy rats (LP, 21 days of gestation, 330 ± 10 g, *n* = 10), and postpartum rats (PP, 7 days, 270 ± 8 g, *n* = 10). All rats were provided with water and food ad libitum and were maintained in individual acrylic cages on a 12 : 12 h light-dark cycle with an average daily temperature of 24 ± 1°C and an average humidity of 60–70%. The day of the experiment, all the rats, previously anesthetized (pentobarbital sodium, 50 mg/kg, intraperitoneally), were sacrificed by cervical dislocation and then the hearts were excised and weighed; subsequently the left ventricle was dissected, immediately frozen in liquid nitrogen, and stored at −80°C for posterior analysis.

### 2.2. Quantitative PCR

Total RNA was isolated individually from frozen left and right ventricle samples using TRIzol reagent (Invitrogen) following the manufacturer's instructions. Genomic DNA in total RNA was eliminated by digestion using recombinant DNase I (Roche, Indianapolis, IN, USA) as specified by the manufacturer. Separate cDNAs from each tissue were synthesized from total DNA-free RNA (1 *μ*g) using the QuantiTect Reverse Transcription kit (Qiagen, Valencia, CA, USA) and oligo-dT (0.5 *μ*g).

Col I, Col III, and glyceraldehyde 3-phosphate dehydrogenase (GAPDH) mRNA were measured by quantitative PCR (qPCR) using the primers shown in Table 1. qPCR reactions were performed for Col I and Col III and normalized by the mRNA expression of GAPDH using 3 *µ*L of cDNA for each gene. Standard curves of Col I, Col III, and GAPDH were run to determine the efficiency of amplification using dilutions from 5*E*10^−4^ to 5*E*10^−8^ ng/*μ*L of PCR fragment. For each measurement, the expression levels (ng/*μ*L) were normalized to GAPDH and expressed as the ratio of Col I or Col III to GAPDH. GAPDH was used as a housekeeping gene; previous studies demonstrated that GAPDH expression in cardiac tissues did not change during pregnancy [5, 17].

### 2.3. Western-Blot Analysis

To evaluate protein expression levels, left ventricular tissue was homogenized with a Polytron homogenizer in lysis buffer and protease inhibitor cocktail (Roche). Fifty micrograms of total protein was separated by 10% SDS-PAGE. Separated proteins were electrotransferred to nitrocellulose membranes. Membranes were blocked with 5% blocking agent (Amersham, GE healthcare) and immunoblotted using the following antibodies: Col I and Col III, and GAPDH was used as a load control (Santa Cruz Biotechnology). After that, a secondary horseradish peroxidase-conjugated antibody was applied for 1 hour at room temperature. The blots were developed with a chemiluminescence detection system (Amersham, GE Heath care) and visualized by exposure to Kodak radiographic film. Density of bands was measured with Image J (National Institutes of Health, Bethesda, MD, USA).

### 2.4. Statistical Analysis

The obtained data were expressed as means ± SEM. To compare two groups Student's *t*-test was used. For comparison of more than two experimental groups, analysis of variance of one factor and post hoc Tukey's test were applied. All the differences were considered statistically significant with a *P* ≤ 0.05. Statistical analysis was conducted with the software Minitab Release version 12.

## 3. Results and Discussion

**Figure 1:**
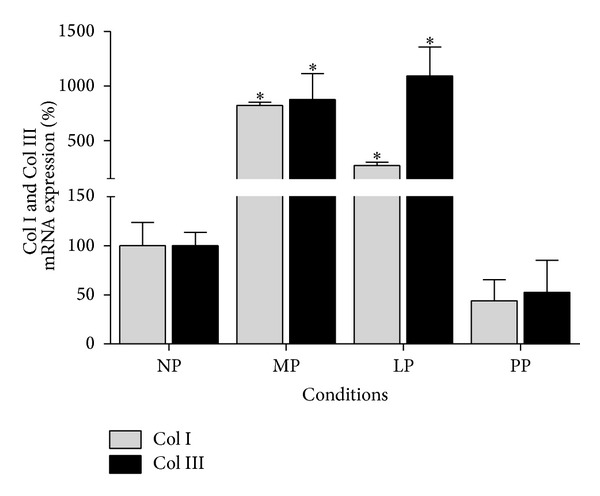
Collagen isoforms mRNA expression in left ventricle during pregnancy and postpartum. NP: nonpregnant group (*n* = 10), MP: midpregnancy group (*n* = 10), LP: late-pregnancy group (*n* = 10), and PP: postpartum group (*n* = 10). Values are expressed as means ± SEM. *P < 0.05 versus NP group.

Col I and Col III mRNA and protein expression levels were quantified in left ventricle from rat heart to determinate their expression in response to pregnancy and postpartum. Col I and Col III transcripts increased (*P* > 0.05) 8.2-fold and 8.8-fold, respectively, after 12 days of pregnancy (see Figure 1). In contrast, after 21 days of pregnancy Col I increased (*P* > 0.05) 2.7-fold and Col III increased 10.9-fold compared to nonpregnant group. In postpartum, Col I and Col III transcripts returned to basal levels (see Figure 1).

**Figure 2:**
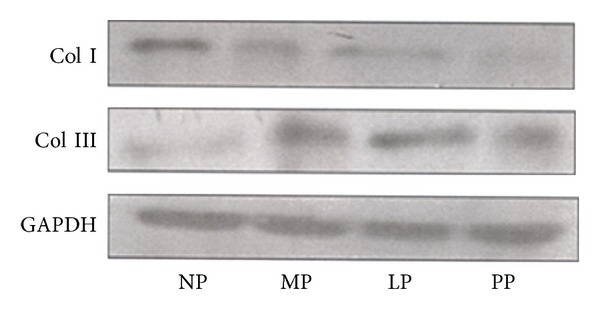
Representative immunoblots. NP: nonpregnant group; MP: midpregnancy group; LP: late-pregnancy group; and PP: postpartum group.

**Figure 3:**
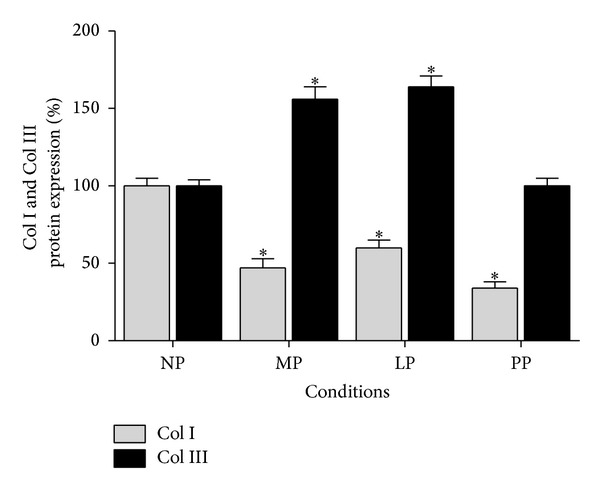
Collagen isoforms protein expression in left ventricle during pregnancy and postpartum. NP: nonpregnant group (*n* = 10); MP: midpregnancy group (*n* = 10); LP: late-pregnancy group (*n* = 10); and PP: postpartum group (*n* = 10). Values are expressed as means ± SEM. *P < 0.05 versus NP group.

On the other hand, the protein expression analysis revealed that Col I expression in left ventricle decreased significantly (*P* > 0.05) during pregnancy (MP and LP groups) and postpartum compared with nonpregnant group (see Figures 2 and 3). Moreover, the results of Col III expression analysis in left ventricle show that, at protein level, an increase (*P* > 0.05) during pregnancy reaching a maximal point of expression in late pregnancy is observed (LP group increased 1.6-fold versus NP group; see Figure 3). The data obtained in this study also show that Col III expression level is similar in nonpregnant and postpartum group (see Figure 3).

Collagens are the main component of cardiac extracellular matrix, which participate in the pathologic heart remodeling [18–21]. However, the Col I and Col III participation in heart remodeling in response to pregnancy is not well defined. In the present study, the Col I and Col III mRNA and protein expression levels in left ventricle from rat heart were determined during pregnancy. Col I and Col III mRNA expression levels are upregulated in left ventricle during pregnancy and reversed during postpartum. The increase in Col I and Col lll mRNA along with differential protein expression suggests that both collagens contribute to heart remodeling during pregnancy. A previous study reported that collagen isoforms gene expression in cardiac tissue did not change during pregnancy [22], while other reports showed that Col III is downregulated in late pregnancy [23]. However, our data clearly show that Col I and Col III mRNA expression levels in left ventricle increased at 12 and 21 days of pregnancy. Also, we show that Col III protein expression in left ventricle increased in late pregnancy while Col I decreased. Some reports have shown that matrix metalloproteinase-2 in heart, which cleaves Col III [24], is downregulated during late pregnancy [25]; this could suggest that a suppression in matrix metalloproteinase-2 expression in heart promotes Col III expression in extracellular matrix. In contrast, other studies using histological analysis have reported a minimal increase in collagen content in the heart during pregnancy [9] or no difference [10, 22].

Therefore, our data suggest that the differential expression of collagen isoforms in heart during pregnancy could play an important role in the regulation of the developed passive tension in myocardial walls during the diastolic filling. The changes in collagen isoforms expression observed in this work could explain the decrease in cardiac stiffness in late pregnancy reported in previous studies [6], suggesting an adaptive mechanism to compensate the demand of blood volume overload in heart during pregnancy.

The mechanism that regulates the collagen expression in heart during pregnancy is still unknown. An early study reported that estrogens reduce the Col I/Col III ratio in age-related left ventricular remodeling; this reduction was result of a decrease in Col I accompanied by an increase in Col III protein expression [26]. It is known that during pregnancy there are characteristic changes in hormonal levels and in early pregnancy the level of progesterone is increased while the level of estrogens is increased in late pregnancy [27]. These changes could be regulating collagen expression, and therefore the mechanism implicated in the control of differential expression of collagen isoforms during pregnancy could be hormonal. Recent studies have shown that, in early pregnancy, progesterone regulates development of cardiac hypertrophy through a calcineurin-dependent pathway [17] and the activation of ERK1/2 [10]. Moreover, estrogens increase MCIP1 (modulatory calcineurin interacting protein 1) expression (a calcineurin inhibitor) [17] suggesting that estrogens inhibit the effect of calcineurin in late pregnancy. However, more experiments should be performed to demonstrate that estrogens and progesterone are also involved in the regulation of the expression of collagen isoforms in cardiac remodeling during pregnancy. Besides, although Col I and Col III mRNA were upregulated in left ventricle; interestingly at protein level, they were differentially expressed in heart during pregnancy, leaving to elucidate the mechanism of posttranscriptional regulation that is involved. Recent reports show that microRNAs (miRNAs) play an important role in the regulation of cardiovascular diseases [28–31]. The miRNAs miR-29 family and miR-133 regulate mRNAs that encode proteins involved in fibrosis during the pathologic cardiac remodeling [32, 33]; however, the participation of these miRNAs in physiologic cardiac remodeling induced by pregnancy is still unknown.

## 4. Conclusions

Collagen is regulated in the physiological cardiac remodeling induced by pregnancy and postpartum. The differential expression of collagen at protein level in left ventricle can act as an important regulation mechanism in the blood volume overload that is present during pregnancy and that is reversed in postpartum.

## Figures and Tables

**Table 1:** Primers used for quantitative PCR analysis.

Gene/primer name	Primer sequence (5′-3′)
Collagen I (Col I)	
Col I-forward	AAGACATCCCTGAAGTCAGC
Col I-reverse	CCTATGACTTCTGCGTCTGG
Collagen III (Col III)	
Col III-forward	ACAGCAGTCCAATGTAGATG
Col III-reverse	GAGCAGGTGTAGAAGGCTG
Glyceraldehyde-3-phosphate dehydrogenase (GAPDH)	
GAPDH-forward	GGACATTGTTGCCATCAACG
GAPDH-reverse	CTCCATGGTGGTGAAGACGC
